# Active Human and Porcine Serum Induce Competence for Genetic Transformation in the Emerging Zoonotic Pathogen *Streptococcus suis*

**DOI:** 10.3390/pathogens10020156

**Published:** 2021-02-03

**Authors:** Maria Laura Ferrando, Alex Gussak, Saskia Mentink, Marcela Fernandez Gutierrez, Peter van Baarlen, Jerry Mark Wells

**Affiliations:** Host-Microbe Interactomics, Animal Sciences, Wageningen University, 6708 WD Wageningen, The Netherlands; laura.ferrando@wur.nl (M.L.F.); alex.gussak@wur.nl (A.G.); saskia.mentink@wur.nl (S.M.); marcela.fernandez@wur.nl (M.F.G.); peter.vanbaarlen@wur.nl (P.v.B.)

**Keywords:** *Streptococcus suis*, natural competence, DNA transformation, virulence, transformasome, serum complement

## Abstract

The acquisition of novel genetic traits through natural competence is a strategy used by bacteria in microbe-rich environments where microbial competition, antibiotics, and host immune defenses threaten their survival. Here, we show that virulent strains of *Streptococcus suis*, an important zoonotic agent and porcine pathogen, become competent for genetic transformation with plasmid or linear DNA when cultured in active porcine and human serum. Competence was not induced in active fetal bovine serum, which contains less complement factors and immunoglobulins than adult serum and was strongly reduced in heat-treated or low-molecular weight fractions of active porcine serum. Late competence genes, encoding the uptake machinery for environmental DNA, were upregulated in the active serum. Competence development was independent of the early competence regulatory switch involving XIP and ComR, as well as sigma factor ComX, suggesting the presence of an alternative stress-induced pathway for regulation of the late competence genes required for DNA uptake.

## 1. Introduction

Natural competence, the process by which bacteria acquire environmental DNA with the potential for homologous recombination in the genome [1], may confer selective advantages to bacterial pathogens for the transmission, colonization, and infection of the host, leading to the emergence of more pathogenic clones [2,3]. 

In some streptococcal species, competence for DNA transformation is a quorum-sensing trait regulated by a secreted peptide pheromone [4]. Stress induced by DNA-damage, nutrient limitation, and exposure to antibiotics has also been shown to promote competence development [5,6,7]. However, the factors that induce competence for DNA transformation in pathogens and commensal species in vivo are largely unknown.

*Streptococcus suis* (SS), a swine pathogen and emerging zoonotic agent, can invade host tissues and spread systemically through the bloodstream to cause sepsis, meningitis, and other sequelae [8]. Serotyping of the capsule polysaccharide is the most widely used method for the epidemiology of invasive *S. suis*. However, the use of serotyping alone as a predictor of virulence has the limitation that strains of the same serotype can vary substantially [8,9]. This reflects the high genetic variability of different strains and the risk for the emergence of novel hypervirulent and zoonotic strains, as well the transmission of antibiotic resistance [10,11]. The high genomic diversity of *S. suis* suggests that high levels of recombination occur between strains in vivo [11]. 

In 2014, our group identified an extracellular pheromone peptide, XIP (*comX*-Inducing Peptide), that induces competence in SS [12]. XIP activates the regulator ComR, which induces the expression of ComX, a sigma factor enabling the specific binding of the RNA polymerase to the promoters controlling the expression of the transformasome competence machinery, including a type IV-like pilus composed of the pilin subunit protein ComYC [12]. Not all SS strains can be transformed by their cognate XIP, including virulent isolates belonging to serotype 7 (SS7) [12]; thus, other methods for inducing competence for DNA transformation would open up possibilities for efficient genetic manipulation. 

Although invasive isolates of SS can grow in active serum, we hypothesized that the stress induced by exposure to complement, SS-binding antibodies and antimicrobial peptides in serum might induce competence for DNA transformation at sites of infection [13]. We investigated (1) competence induction in active and heat-inactivated porcine and human serum, and (2) the role of early and late regulatory genes *comR* and *comX*, on competence development in active serum.

## 2. Methods

### 2.1. Bacterial Strains, Plasmids, and Culture Conditions

The bacterial strains and plasmids used in this study are listed in Table S1. SS strains were grown in Todd Hewitt Broth (THB; Oxoid, Hampshire, UK) supplemented with 0.2% yeast extract (Difco; THY) at 37 °C with 5% CO_2_ porcine serum (PS; cat. 26250084, Gibco). Fetal bovine serum (FBS; cat. FBS-12A, Capricorn) was slowly defrosted at 4 °C overnight (O/N), split in aliquots of 30 mL, and stored at −20 °C for extended use. Human serum (HS) was derived from blood that was obtained from three healthy volunteers via Sanquin. The blood was allowed to clot for 15 min at room temperature, the serum was collected after centrifugation for 10 min at 4000× *g* at 4 °C, and was pooled and subsequently stored at −20 °C. Heat-inactivated serum was prepared by incubation for 30 min at 56 °C with gentle shaking. When necessary, antibiotics were added to the culture media at the following concentrations: chloramphenicol (Cm) 5 µg/mL and spectinomycin (Spc) 100 µg/mL. 

### 2.2. RNA-Seq

Bacterial cultures were grown in triplicate to the mid-exponential phase in 10 mL of THY medium or serum. After centrifugation, the bacterial pellet was resuspended in a QIAzol buffer (Qiagen, Hilden, Germany), added to vials containing silica beads, and lysed using FastPrep-24 5G (MP Biomedicals, Santa Ana, CA, USA; settings: 4.0 m/s and run time 40 s). The bacterial lysate was centrifuged for 5 min at 16.000× *g* and the supernatant stored at −80 °C or was immediately used for RNA isolation using the miRNeasy mini kit (Qiagen). The RNA quantity was determined using the Qubit 4 Fluorometer and Nanodrop spectrophotometer (Thermo Fisher, Waltham, MA, USA). The RNA quality, expressed as the RNA integrity number (RIN) was determined by electrophoresis using the 2200 TapeStation system (Agilent, Santa Clara, CA, USA). Strand-specific libraries were prepared for Illumina mRNA sequencing (RNA-seq) by Novogene Bioinformatics Technology Co., Ltd., in Hong Kong, China. Microbial rRNA was first removed using the Illumina Ribo-zero kits prior to the synthesis of single stranded cDNAs essentially as described [14]. Double stranded cDNAs were end-repaired, polyadenylated, and ligated with adapter sequences before size-selection using AMPure XP beads (Beckman Coulter, Brea, CA, USA). The uracil containing cDNA strands were degraded by a Uracil-Specific Excision Reagent (USER) enzyme, and the remaining DNA was amplified by PCR and purified using AMPure XP beads. The library DNA concentration was first quantified using a Qubit 2.0 fluorometer (Life Technologies, Carlsbad, CA, USA), and then diluted to 1 ng/µL before checking the insert size on a 2100 Bioanalyzer instrument (Agilent). 

Sequencing was performed using the Hiseq Illumina PE150 platform. FASTQ reads were analyzed using CLC Genomics Workbench (GB) 11.0 (Qiagen). After trimming and quality control, the reads were mapped against the SS2 P1/7 reference genome (NCBI Nucleotide accession no. AM946016). The expression levels were normalized as transcripts per million (TPM) using the RNA-seq analysis option in CLC GB. For identification of the differentially expressed transcripts, we used a two-tailed *t*-test with adjusted *p*-values using a false discovery rate (FDR). Genes with adjusted *p*-values below 0.05 were considered significantly differentially expressed and values were presented as log_2_ fold-change. The RNA-seq data have been deposited at https://easy.dans.knaw.nl/ui/datasets/id/easy-dataset:159795.

### 2.3. Standard Assay for DNA Transformation

To establish a standard method for testing the competence for DNA transformation in active porcine serum (aPS), we made 10-fold, 100-fold, and 1000-fold dilutions of an overnight (ON) culture of SS P1/7 in THY in 30 mL active porcine serum (aPS; Figure S1). At hourly intervals, 200 µL was transferred into a new 1.5 mL Eppendorf tubes containing 10 µL of transforming DNA at a concentration of 200 ng/µL or sterile water (used as a control), and incubated for a further 2 h at 37 °C. Aliquots of the culture were then plated on THY agar plates containing the required antibiotic and were incubated at 37 °C for 1 to 2 days. Based on these results, the subsequent competence assays were mostly performed with a 10-fold dilution of an ON SS culture, giving an initial of OD_600nm_ of approximately 0.08. the bacterial growth in the serum was measured by spectrophotometry at OD_600nm_ (SpectraMax M5) with a 1:1 dilution in PBS to compensate for the high turbidity of the serum.

As the transforming DNA, we used plasmid pNZ8048 (3.2kb) conferring resistance to Cm. To confirm transformation with plasmid pNZ8048, a colony PCR was performed on chloramphenicol-resistant colonies using primers *cm*_F and *cm*_R (Table S1). In one experiment, a linear DNA fragment was used for the DNA transformation. This fragment was amplified from the genomic DNA of an S10 Δ*apuA* mutant, in which the *apuA* gene was interrupted by a spectinomycin (Spc) resistance gene [15] (see details in Figure S4). A homologous recombination of the amplicon in the genome of SS P1/7 was detected by colony PCR using primers *apuA*_F and *apuA*_R (Table S1 and Figure S4).

In some assays, modifications were made to the serum or culture medium, as follows: a low molecular weight (MW) serum fraction containing short peptides that might resemble the natural competence-inducing peptide (XIP) was obtained by centrifugal ultrafiltration of 20 mL aPS at 8000× *g* for 1 h using a 10 kDa cut-off filter (Vivaspin^®^ 20 (Sartorius, Göttingen, Germany). The effect of long-term storage of aPS was evaluated by storing the serum at 4°C for 14 days before the assay was conducted. Overnight THY precultures and aPS were also supplemented with 100 µM Iron (Fe^II^) sulfate heptahydrate [16] (D9533-1G, Sigma) or depleted of free iron through the addition of the chelator deferoxamine mesylate salt (DIF). Iron (Fe^II^) sulfate heptahydrate and DIF were also added to THY as controls. Finally, competence for DNA transformation was tested in complex media (CM) [15] alone, or CM supplemented with glucose as a carbon source.

## 3. Results and Discussion

An analysis of the RNA-seq data identified 1503 significantly differentially expressed genes, of which 745 were increased and 758 decreased in aPS compared with THY (Figure S2). Many of the reported SS virulence genes (45 out of 160) [17] were differentially regulated in the serum compared with THY (Figure S3). In THY, the late competence genes ComYA to ComYH encoding the DNA uptake and translocation machinery [12] were either not expressed or only transcribed in low amounts, with the exception of *comYH*. However, transcription of the late competence operon was upregulated in aPS, except for *comYH*. Transcription of *comX* encoding the sigma factor regulating the expression of the late competence operon was decreased ~0.59-log_2_ fold in the serum compared with THY (Table 1). Furthermore, the expression of the genes encoding the proteins involved in ssDNA binding and homologous DNA recombination was significantly decreased in the serum compared with THY (Table 1). 

**Figure 1:**
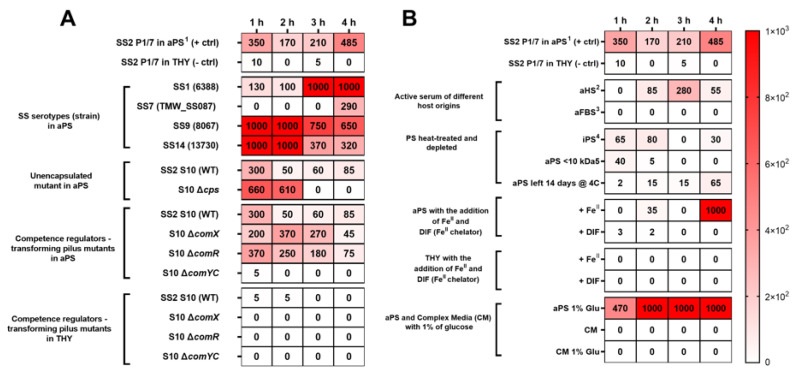
Heatmap showing transformants/mL of *Streptococcus suis* (SS) recovered during the induction of competence for DNA transformation in aPS and other media in the presence of 2 µg of plasmid. The top row of each panel shows the average number of transformants/mL of SS2 P1/7 in aPS (used as a positive control (+ctrl)) and SS2 P1/7 in THY (negative control (−ctrl)) included in different experiments. (**A**) The transformation efficiencies of different SS strains: SS serotypes grown in aPS; DNA transformation efficiency of SS2 strain S10 (WT) and isogenic unencapsulated mutant SS2 S10 Δcps grown in aPS; and DNA transformation efficiency of mutants of the natural competence regulators ComR/ComX and mutant of ComYC DNA-binding pilus in SS2 strain S10 grown in aPS or THY. (**B**) Transformation efficiency of SS2 P1/7 grown in different serums or mediums (aPS1—active porcine serum; aHS2—active human serum; aFBS3—active fetal bovine serum; iPS4—inactive porcine serum at 56°C for 30 min; aPS < 10 kDa5 = serum depleted of serum components > 10 kDa; CM—complex medium; Glu—glucose *w*/*v*).

These results prompted us to test SS competence for DNA transformation in aPS (Figure 1). Transformation efficiencies up to 1.0 × 10^3^/mL were obtained with six different SS strains [9] grown for 1 to 4 h in aPS (Figure 1A). This included SS7 strain TMW_SS087, which could not be transformed using the cognate XIP allele found in its genome [12]. The deletion of the capsule genes (∆*cpsEF*) appeared to have an influence on the transformation efficiency (Figure 1). The DNA transformation efficiencies obtained through the growth of SS in active serum were substantially higher than those obtained by growth for 14 h in THY or complex medium, which yielded between 0 and 10 transformants/mL (Figure 1). In these experiments, transformation with plasmid DNA was confirmed by the colony PCR of putative transformants (Figure S4). The differences in the transformation efficiency are unlikely to be related to the cell density or growth rate, as the absorbance values (OD_600nm_) of the SS grown in the porcine and human serum and heat-inactivated serum were similar to the ODs obtained in the culture in THY for the first 3 h of growth (Figure S5).

The transformation efficiency of SS in the active serum was lower than that reported using the XIP pheromone for inducing natural competence in THY, with optimal conditions giving ~1 × 10^6^ transformants/µg [12]. This led us to hypothesize that the induced competence for DNA transformation observed by growth in the active serum might be independent of ComR and ComX. The transformation efficiencies obtained with the WT SS2 strain 10 and the Δ*comR* or Δ*comX* mutants [12] were similar, indicating that serum-induced competence was indeed independent of ComR-XIP regulation and the sigma factor ComX, which is required for XIP-induced competence in laboratory medium [12]. However, a *comYC* mutant that is unable to produce pili grown in aPS was not transformable, highlighting the importance of the transforming pilus for DNA uptake and transformation (Figure 1A). 

We tested the transformation efficiency using the active serum of different hosts. The growth of SS2 strain P1/7 in active human serum (aHS) also induced competence for DNA transformation, with efficiencies of ~2.8 × 10^2^ transformants/mL after 3 h of incubation (Figure 1B). In contrast, growth in fetal bovine serum (aFBS), which contains lower levels of complement proteins and antibodies than the serum from adult bovines, appeared to abolish competence for DNA transformation (Figure 1B). No transformants were observed in any of the three tested dilution ratios (10-fold, 100-fold, and 1000-fold), indicating that competence development was not related to cell density or growth phase (not shown). To gain insight into the mechanisms increasing SS competence for DNA transformation, we modified the standard DNA transformation assay. Firstly, we inactivated the complement activity of porcine serum by heating it at 56 °C for 30 min, which reduced the transformation efficiencies (Figure 1B). Transformation efficiencies were also reduced using aPS stored for 14 days at 4 °C, or using a filtrate of aPS containing only solutes and polypeptides with a molecular weight less than 10 kDa (Figure 1B). To determine whether the limited amount of free iron in the serum might induce competence, we added 100 µM iron (Fe^II^) sulphate heptahydrate to aPS. This did not increase the transformation efficiency, whereas the addition of the iron chelating agent DIF [16,18] appeared to inhibit competence for DNA transformation (Figure 1B). The addition of 1% glucose to aPS appeared to increase the transformation efficiency compared with the positive control, whereas no transformants were recovered in CM or CM + 1% glucose (Figure 1B).

To test whether serum-induced DNA transformation could occur via genome recombination with homologous linear DNA, we used a PCR-amplified DNA fragment containing the *apuA* gene interrupted by a spectinomycin-resistance gene [15]. Out of 20 Spc^r^ transformants tested through PCR, one colony had the correct insertion of the *apuA::spc* fragment into the *apuA* gene (Figure S4). The low frequency of recombination may be due to the high rate of spontaneous resistance to spectinomycin [19]. To evaluate these results in a more natural situation, we showed that DNA transformation was possible with a bacterial lysate of the *apuA* mutant strain (not shown).

We conclude that competence for DNA transformation can be induced in potentially zoonotic SS2 strains when cultured in aHS or aPS. Indeed, multiple genetically diverse SS strains and serotypes became naturally transformable with plasmid or linear DNA in aPS, including one strain that could not be transformed with its cognate XIP peptide under the conditions described by Zaccaria et al. [12].

In aPS, DNA transformation occurred independently of the production of ComR, indicating that XIP or XIP-like peptides were not required for the expression of the late competence operon in aPS. The mechanism was also independent of *comX,* encoding the sigma factor that is required for XIP-induced expression of the late competence genes and the induction of competence for DNA transformation [12,20]. These findings suggest that other regulatory mechanism(s) induced the expression of the late competence genes involved in DNA uptake during the culturing of SS in the active serum. 

Natural competence may allow for the uptake of foreign DNA at infection sites and contribute to bacterial survival and virulence [21]. In infected mucosal tissues, the leakage of plasma from the microvasculature is a hallmark of inflammation and may therefore increase DNA uptake by SS. In addition, we recently showed that the intestinal epithelium of organoids expresses complement factors [22], and that inflammatory stimuli increase the expression of the complement factors involved in the early opsonization and chemotaxis of phagocytes [23]. Although SS possesses mechanisms to avoid complement mediated lysis [24,25], complement may induce a stress response, leading to the induction of competence and DNA uptake from lysed bacteria through an unknown mechanism. The induction of competence could be advantageous, as it enables the integration of foreign DNA, increasing genetic variation within the population, and at low frequencies, foreign DNA might confer a selective advantage to the recipient bacteria. The RNA-seq transcriptome analysis of serum grown SS revealed a high expression of genes involved in the general stress response (universal stress protein UspA log_2_ fold change = 5.4) and DNA repair system (MutT-SSU0251 = 4.6). The primary function of the UspA superfamily is to protect DNA and bacteria against superoxide stress during exponential growth [26]. 

Apart from the relevance of this finding to pathogen evolution, it also provides a simple and easy method for the transformation of SS. This can be useful for strains containing different alleles of the XIP, and strains that may have lost the genes required for XIP induced competence.

## Figures and Tables

**Table 1:** Differential gene expression, shown as log_2_ fold-change values, of SS2 strain P1/7 grown in active porcine serum (aPS) compared with Todd Hewitt broth supplemented with 0.2% yeast extract (THY)—the standard growth media.

SS2 P1/7 Locus Tag	Protein	Functions	aPS vs. THY (Log_2_ Fold Expression Change)
SSU0049	ComR	Transcriptional activator of ComX	−2.42
SSU0016	ComX	Master transcriptional regulator of the transformasome	−0.59
SSU0061	CinA	DNA binding and homologous recombination	−0.31
SSU0062	RecA		−0.70
SSU0924	RadC		−0.52
SSU1083	CoiA		0.66
SSU0126	ComYA	Transformasome apparatus	4.02
SSU0127	ComYB		4.72
SSU0128	ComYC		5.48
SSU0129	ComYD		4.91
SSU0130	ComYE		3.05
SSU0131	ComYF		4.20
SSU0132	ComYG		4.26
SSU0133	ComYH		−2.51
SSU0144	SsbB	ssDNA binding and protection	−1.34
SSU0610	ComEA	dsDNA receptor and channel	2.07
SSU0611	ComEC		2.33

## Data Availability

The RNA-seq data presented in this study are openly available at https://easy.dans.knaw.nl/ui/datasets/id/easy-dataset:159795.

## Supplementary Materials

The following are available online at https://www.mdpi.com/2076-0817/10/2/156/s1. RNA-seq data were deposited at https://easy.dans.knaw.nl/ui/datasets/id/easy-dataset:159795. Table S1: strains and plasmids used in this study. Figure S1: Scheme for establishing optimal conditions for DNA transformation in aPS. Figure S2: Overview of the RNA-seq data. Figure S3: Virulence genes divided based on their known role in virulence and competence genes differentially expressed in SS2 grown in active porcine serum. Figure S4: PCR verification of DNA transformants. Figure S5: OD_600nm_ measurement of SS2 strain P1/7 growth in different sera and media.
