# Supplementary material for: Active Human and Porcine Serum Induce Competence for Genetic Transformation in the Emerging Zoonotic Pathogen Streptococcus suis
Source: Pathogens. 2021 Feb 3;10(2):156. doi: 10.3390/pathogens10020156 (PMC7913127; doi:10.3390/pathogens10020156)
Supplement: Supplementary file 1 [file pathogens-10-00156-s001.pdf]

Supplementary Information

Table S1. Strains and plasmids used in this study.

Strain	Serotype	SS2 P1/7 locus Tag	Description	Ref.
P1/7	2		Virulent strain SS2 isolated from diseased pig in the UK	[1]
TMW_SS087	7		Virulent strain SS7 isolated from diseased pig in the UK	*This study*
8067	9		Virulent strain SS9 isolated from diseased pig in the NL	[1]
S10	2		Virulent strain SS2 S3881	[1]
6388	1		Virulent strain SS1	[1]
13730	14		Virulent strain SS14	[1]
S10 *ΔcomX*	2	SSU0016	*comX*::*spc* mutant of strain S10	[2]
S10 *ΔcomR*	2	SSU0049	*comR*::*spc* of S10	[2]
S10 *ΔcomYC*	2	SSU0128	*comYC*::*spc* of S10	[2]
S10 *ΔapuA*	2	SSU1849	*apuA::spc* of S10	[3]
S10 *ΔcpsEF*		SSU0519/ SSU0520	Deletion of *cpsE/F* capsular genes of S10	[1]
**Plasmid**				
pNZ8048			*E. coli* and *L. lactis* shuttle vector containing PnisA promoter, Cm^R (chloramphenicol resistant) size 3.2 kb	[2]
**Primers**			Sequence (5′- 3′)	
*cm*_F			actggttacaatagcgacgga	
*cm*_R			acccagtaaatgaagtccaagga	
*apuA*_F			agacgcttgagctgttggag	
*apuA*_R			caatagcaagaagccaatgga	
Δ*apuA* _F			gccaagaagttgcctggttg	
Δ*apuA* _R			tattgcgggaaatgcagtgg	
*spc*_F			ggatcaggagttgagagtggac	
*spc*_R			ccactgcatttcccgcaata	

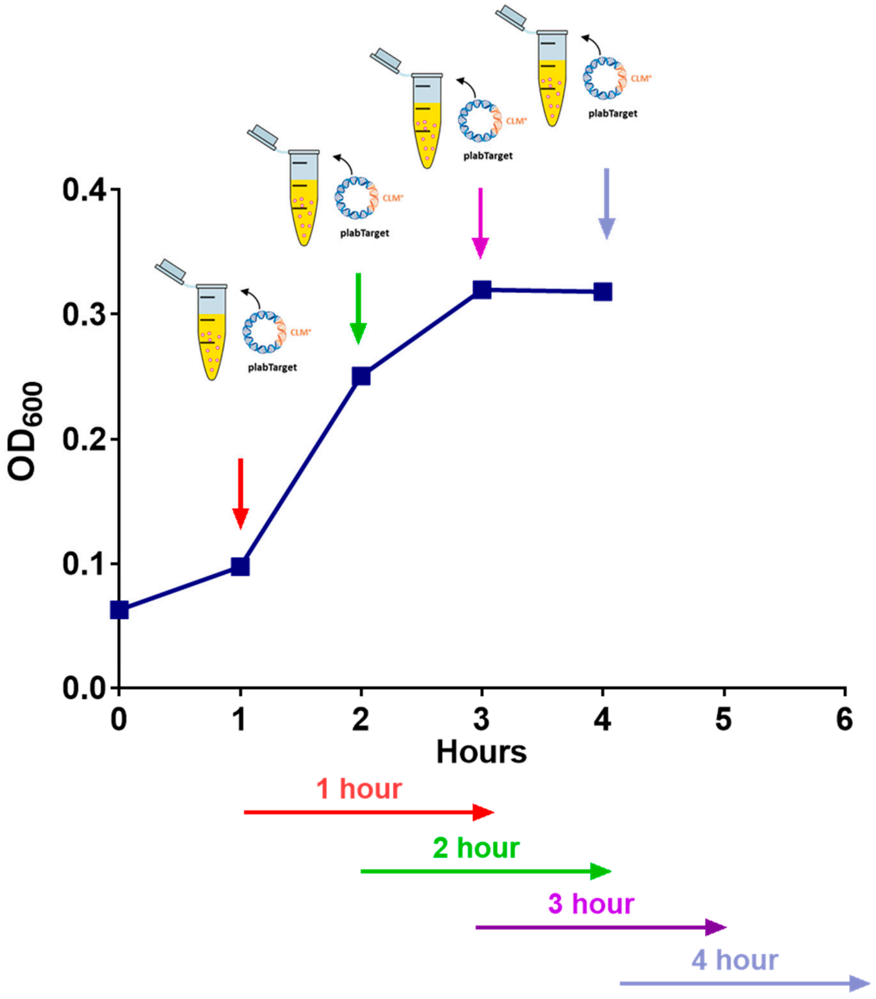

Figure S1. Scheme for establishing optimal conditions for DNA transformation in active porcine serum (aPS). Overnight, Todd Hewitt broth supplemented with 0.2% yeast extract (THY) culture of SS2 strain P1/7 was diluted 10-fold in serum and incubated for 4 h. Every hour, 200 µl of bacterial subculture in serum was transferred into 1.5 ml Eppendorf tubes, and 2 µg of plasmid was added to the subculture that was successively incubated at 37 ^{0}C for two additional hours. After that, the subculture was plated on selective THY agar plates at 37 ^{0}C for 1 day. Colonies were screened for the presence of the plasmid using colony PCR, as shown in Figure S5.

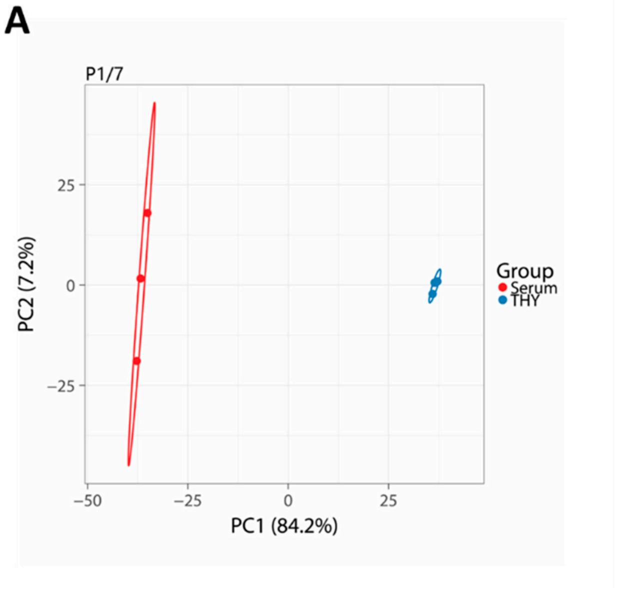

Figure S2. Overview of the RNA-seq data: (A) PCA plot showing perfect separation between RNA samples extracted from *S. suis* grown in THY (blue dots) or active porcine serum (red dots). (B) Volcano plot displaying differentially expressed genes. Blue dots represent downregulated genes and red dots represent upregulated genes.

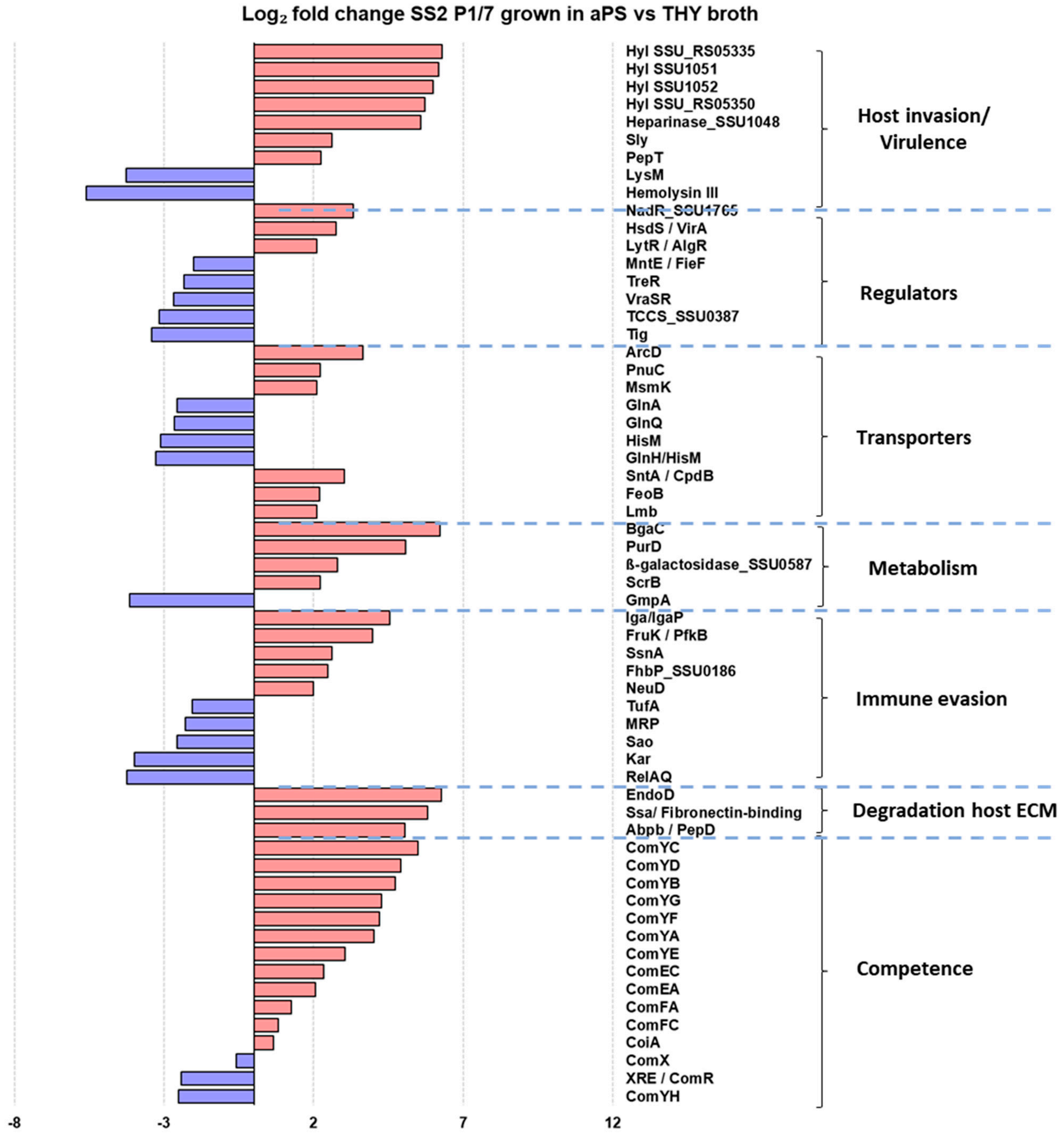

Figure S3. Virulence genes grouped by their role in virulence [4], and competence genes differentially expressed in SS2 grown in active porcine serum (aPS). Pink and violet bars indicate the up- and down-regulation of the gene expressions, respectively, in aPS compared with the THY media. Hyl—Hyaluronate lyase, SSU1050; Heparinase_SSU1048—Heparinase II/III-like protein, SSU1048; Sly—suilysin, SSU1231; PEP T—Peptidase T, SSU0973; LysM—surface protein containing a LysM at the N-terminus, SSU0215; hemolysin III, SSU0854; HsdS/VirA—restriction endonuclease S subunit, SSU1589; NadR—transcriptional regulator, SSU1765; LytR/AlgR—putative DNA-binding protein, SSU1907; MntE/FieF—manganese efflux system, SSU0984; TreR—trehalose operon repressor, SSU0218; VraS/VraR—two-component system, SSU0388; TCCS_SSU0387—two-component system sensor histidine kinase, SSU0387; Tig—trigger factor, SSU0306; ArcD—arginine deiminase system, SSU0584; PnuC—nicotinamide mononucleotide transporter, SSU1764; MsmK—multiple sugar-binding transport ATP-binding protein, SSU1701; GlnA—hypothetical protein, SSU0157; GlnQ—putative glutamine transporter, ATP-binding 4, SSU1192; HisM—amino acid ABC transporter permease, SSU0501; GlnH/HisM—glutamine ABC transporter, SSU0883; SntA/CpdB—streptococcal heme-binding protein, SSU1879; FeoB—Fe2+ transport system protein B, SSU1237; Lmb—lipoprotein 103/laminin binding protein, SSU0308; BgaC—β-galactosidase, SSU0402; PurD—phosphoribosyl amine-glycine ligase, SSU0032; ScrB—sucrose-6-phosphate hydrolase, SSU1619; GmpA—phosphoglycerate mutase,

SSU1451; Iga/IgaP—IgA1 protease/zinc metalloprotease, SSU1773; FruK/PfkB—1-phosphofructokinase, SSU0767; SsnA—surface-anchored DNA nuclease, SSU1760; FhbP_SSU0186—fibrinogen binding protein/HP0197/ssPspC, SSU0186; NeuD—sialic acid transferase, SSU0537; TufA—translation elongation factor EF-Tu, SSU0482; MRP—muramidase-released protein, SSU0706; Sao—surface antigen SP1, SSU1201; Kar—3-ketoacyl-ACP reductase, SSU1603; RelAQ—(p)ppGpp/(p)ppGpp synthetase, SSU0916; EndoD—endo-β-N-acetylglucosaminidase D, SSU1715; Ssa—fibronectin-binding/surface-anchored zinc carboxypeptidase, SSU1143; Abpb/PepD—πeptidase C69/Amylase-binding protein B, SSU1215; ComYA—competence protein CglA, SSU0126; ComYB—competence protein CglB, SSU0127; type II ComYG—minor pseudopilin PulG, SSU0132; ComYC—competence protein CglA, SSU0128; ComYD—competence protein CglB, SSU0129; ComYE—competence protein ComGC, SSU0130; ComYF–competence protein CglD, SSU0131; ComFA—SSU0393; ComFc—competence protein Fc, SSU0394; ComEA—competence protein CelA, SSU0610; ComEC—competence protein ComEC, SSU0611; CoiA–competence protein CoiA, SSU1083; ComX—transcriptional regulator ComX, SSU0016; XRE/ComR—gene homologous to *S. mutans* SMU_61, SSU0049; ComYH—competence protein ComGF, SSU0133.

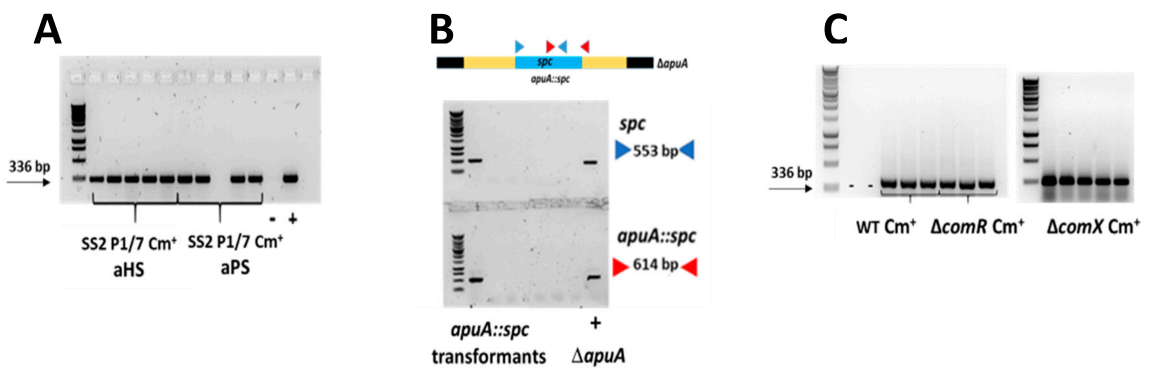

Figure S4. PCR verification of DNA transformants. (**A**) The colony PCR of Cm^R colonies was used to verify the uptake of exogenous plasmid DNA (Cm^+ gene 336 bp) by SS2 P1/7 after growth in aPS or aHS. + is the control plasmid DNA; - is the water control. (**B**) Schematic representation of the homologous recombination of a 3.8 kb PCR fragment containing the spectinomycin resistance gene inserted in the *apuA* gene [3]. Triangles represent the two sets of primers used for the detection of the disrupted *apuA* gene in the recovered colonies. The 614 bp amplicon indicates the recombination of the linear fragment in the genome**.** (**C**) Colony PCR of Cm^R colonies was performed to verify the uptake of exogenous plasmid DNA by SS2 S10 WT and SS2 S10 Δ*comR*/Δ*comX* mutant strains.

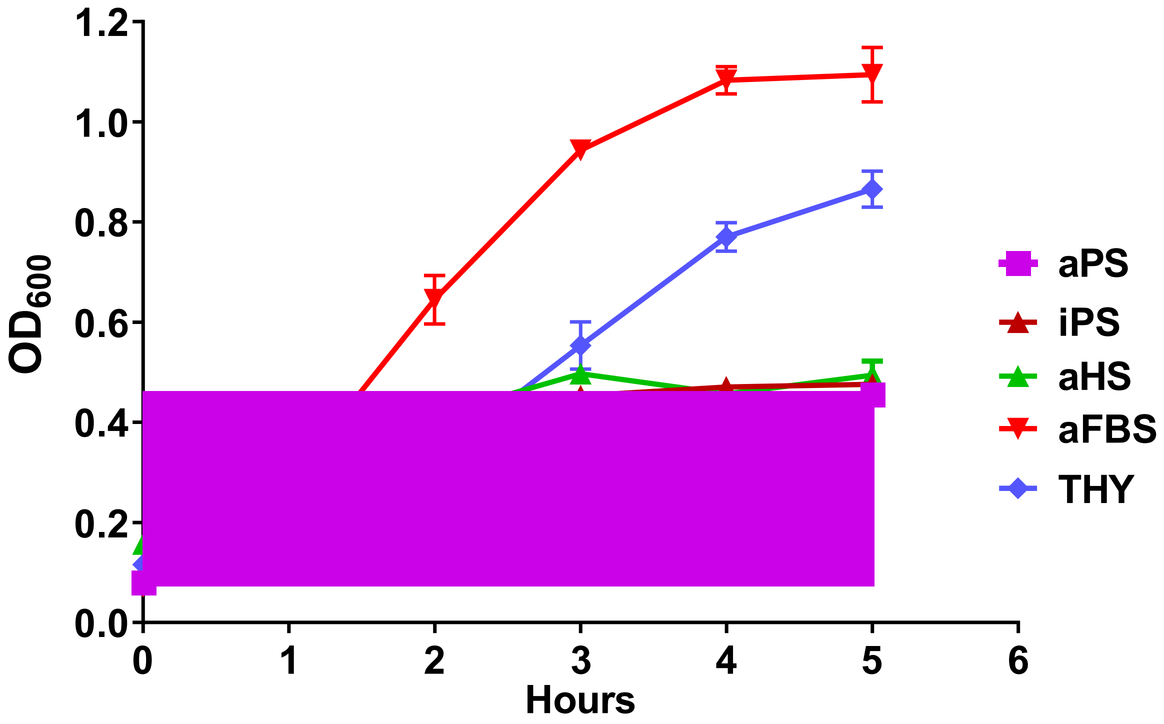

Figure S5. OD_{600} measurements of SS2 strain P1/7 growth in different sera and media. Growth curves represent the average of at least two experiments. aPS—active porcine serum; iPS—inactive porcine serum; aHS—active human serum; aFBS—active fetal bovine serum; THY—Todd Hewitt broth supplemented with 0.2% yeast extract.

References

1. Ferrando, M.L.; de Greeff, A.; van Rooijen, W.J.M.; Stockhofe-Zurwieden, N.; Nielsen, J.; Wichgers Schreur, P.J.; Pannekoek, Y.; Heuvelink, A.; van der Ende, A.; Smith, H.; et al. Host-pathogen Interaction at the Intestinal Mucosa Correlates With Zoonotic Potential of Streptococcus suis. *J. Infect. Dis.* **2015**, *212*, 95–105, doi:10.1093/infdis/jiu813.

2. Zaccaria, E.; van Baarlen, P.; de Greeff, A.; Morrison, D.A.; Smith, H.; Wells, J.M. Control of Competence for DNA Transformation in *Streptococcus suis* by Genetically Transferable Pherotypes. *PLoS One* **2014**, *9*, e99394, doi:10.1371/journal.pone.0099394.
3. Ferrando, M.L.; Fuentes, S.; de Greeff, A.; Smith, H.; Wells, J.M. ApuA, a multifunctional α-glucan-degrading enzyme of *Streptococcus suis*, mediates adhesion to porcine epithelium and mucus. *Microbiology* **2010**, *156*, 2818–2828, doi:10.1099/mic.0.037960-0.
4. Segura, M.; Fittipaldi, N.; Calzas, C.; Gottschalk, M. Critical *Streptococcus suis* Virulence Factors: Are They All Really Critical? *Trends Microbiol.* 2017.
